# The effect of renin–angiotensin–aldosterone system inhibitors on organ-specific *ace2* expression in zebrafish and its implications for COVID-19

**DOI:** 10.1038/s41598-021-03244-5

**Published:** 2021-12-08

**Authors:** Gha-hyun J. Kim, Adam Melgoza, Fei Jiang, Su Guo

**Affiliations:** 1 Department of Bioengineering and Therapeutic Sciences and Programs in Biological Sciences and Human Genetics, University of California, San Francisco, San Francisco, CA 94158 USA; 2 Graduate Program of Pharmaceutical Sciences and Pharmacogenomics, University of California, San Francisco, San Francisco, CA 94158 USA; 3 Department of Epidemiology and Biostatistics, University of California, San Francisco, San Francisco, CA 94158 USA

**Keywords:** Gene expression, Hypertension, Pharmacogenetics, Animal physiology

## Abstract

Among cases of SARS-CoV-2 infections that result in serious conditions or death, many have pre-existing conditions such as hypertension and are on renin–angiotensin–aldosterone system (RAAS) inhibitors. The angiotensin-converting-enzyme-2 (ACE2), a key protein of the RAAS pathway, also mediates cellular entry of SARS-CoV-2. RAAS inhibitors might affect the expression levels of *ace2*, which could impact patient susceptibility to SARS-CoV-2. However, multi-organ-specific information is currently lacking and no species other than rodents have been examined. To address this knowledge gap, we treated adult zebrafish with the RAAS inhibitors aliskiren, olmesartan, and captopril for 7 consecutive days and performed qRT-PCR analysis of major RAAS pathway genes in the brain, gill, heart, intestine, kidney, and liver. Both olmesartan and captopril significantly increased *ace2* expression in the heart, gill, and kidney. Olmesartan also increased *ace2* expression in the intestine. Conversely, aliskiren significantly decreased *ace2* expression in the heart. Discontinuation of compound treatments for 7 days did not return *ace2* expression to baseline levels. While potential risks or benefits of antihypertensive RAAS inhibitors to SARS-CoV-2 infections in humans remain uncertain, this study provides new insights regarding the impact of RAAS inhibitors on organ-specific *ace2* expression in another vertebrate model, thereby providing comparative data and laying scientific groundwork for future clinical decisions of RAAS inhibitor use in the context of COVID-19.

## Introduction

As a global pandemic, the Coronavirus disease 2019 (COVID-19) caused by severe acute respiratory syndrome coronavirus 2 (SARS-CoV-2) has affected more than 140 million people and led to more than 3 million deaths worldwide. SARS-CoV-2 is an RNA virus that spreads and mutates rapidly. Although continued safety guidance and vaccination efforts have played an immense role in controlling the disease, it is likely to remain as a global pandemic for the foreseeable future. The clinical manifestations of SARS-CoV-2 are predominantly respiratory symptoms but some hospitalized patients also suffer from cardiac dysfunctions including myocardial injury, heart failure, and dysrhythmias^1^. Furthermore, studies have shown that hypertension is associated with increased risk of developing COVID-19 complications and increased mortality from COVID-19^2^.

The angiotensin-converting-enzyme-2 (ACE2) is a coreceptor for cellular entry of SARS-CoV2^3^. It is also a key component of the renin angiotensin system (RAAS). ACE2 negatively regulates the Angiotensin II receptor 1 activity by decreasing the ligand Angiotensin II thereby exerting organ-protective effects^4,5^. Many patients with hypertension and cardiovascular comorbidities are commonly prescribed with anti-hypertensive RAAS inhibitors, such as the angiotensin-converting enzyme inhibitors (ACE-Is) and angiotensin II receptor blockers (ARBs). Interest has grown on understanding whether the use of ACE-Is and ARBs can provide potential benefit or harm to COVID-19 patients. So far, clinical studies remain inconclusive regarding the relationship between RAAS inhibitors and outcomes in COVID-19 patients^6^. Animal studies aimed at determining how RAAS inhibitors might affect tissue *ace2* levels have primarily used rodents and focused on restricted tissues (e.g., heart and kidney)^7^, leaving it unclear how these drugs may exert their effects in a broader set of organs and species. The impact of discontinuation of RAAS inhibitors on *ace2* expression has not been evaluated.

In this study, we examined the effects of a panel of antihypertensive drugs (Table 1) on the transcript levels of *ace2* and other RAAS pathway genes across six organs (brain, gill, heart, kidney, intestine, and liver) in adult zebrafish. Previous analysis of single cell transcriptomic data of zebrafish embryos has detected major RAAS pathway genes similar to humans^8^. Zebrafish as a vertebrate possess a high degree of genetic, physiological, and morphological similarity to humans^9–11^. Approximately 71% genes and 84% disease-associated genes are shared between zebrafish and humans^12^. Drugs can be conveniently delivered systemically through direct dissolution in the tank water. Our results have uncovered drug- and organ-specific effects on *ace2* transcript levels and provide a critical comparative dataset in a new species.

**Table 1:** Description of the anti-hypertensive drugs evaluated in this study.

Drug	Pharmaceutical class	Mechanism of action	IC50	MW (g/mol)	Therapeutic indication
Aliskiren	Direct renin inhibitor	Renin inhibitor blocking the conversion of angiotensinogen to angiotensin I	1.5 nM	609.79	Hypertension
Captopril	Angiotensin converting enzyme inhibitor	Blocks the conversion of angiotensin I to angiotensin II	6 nM	217.29	Hypertension, congestive heart failure, diabetic nepropathy
Olmesartan Medoxomil	Angiotensin II receptor antagonist	Selective binding to angiotensin I receptor for competitive blocking of angiotensin II	66.2 μM	558.59	Hypertension, heart failure
Amlodipine besylate	Calcium channel blocker	Block the voltage-dependent L-type calcium channels to inhibit the influx of calcium	1.9 nM	567.05	Angina, coronary artery disease, hypertension

## Results

### Quantitative analysis of RAAS pathway gene expression in six organs reveals tissue-specific enrichment of ace2 transcripts

**Figure 1:**
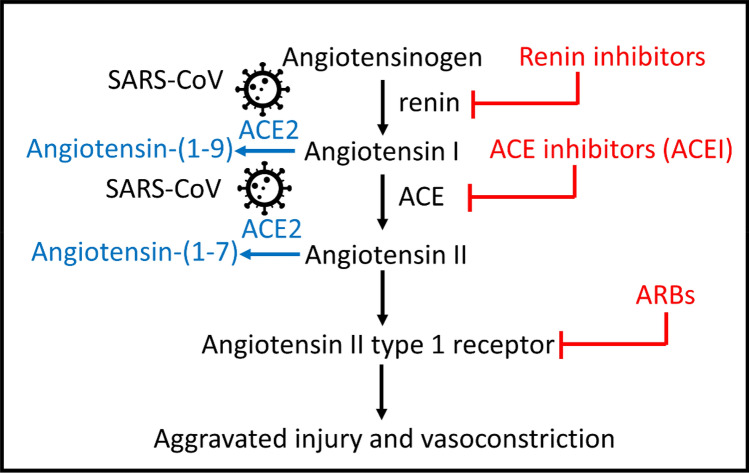
The RAAS pathway and its relationship to SARS-CoV(1 and 2) viruses. Upon entry to cells, the SARS-CoV is known to bind to its functional receptor, angiotensin converting enzyme 2 (ACE2). In normal physiology, renin cleaves angiotensinogen, produced by the liver which yields angiotensin I. The angiotensin converting enzyme (ACE) converts angiotensin I to angiotensin II and the angiotensin II binds to the angiotensin II type 1 receptor which leads to vasoconstriction, aggravated tissue injury and hormonal production. ACE2 cleaves angiotensin II into angiotensin (1–7) to attenuate the effects of vasoconstriction. Another function of ACE2 involves cleaving angiotensin I to angiotensin-(1–9) for the hydrolysis of peptides such as apelin-1. Aliskiren is a direct renin inhibitor that blocks the conversion of angiotensinogen to angiotensin I. Captopril is an ACE-I that blocks the conversion of angiotensin I to angiotensin II. Olmesartan is an ARB ACE-I: Angiotensin Converting Enzyme Inhibitor, ARB: Angiotensin Receptor Blocker, ACE: Angiotensin Converting Enzyme-1, ACE2: Angiotensin Converting Enzyme-2.

**Figure 2:**
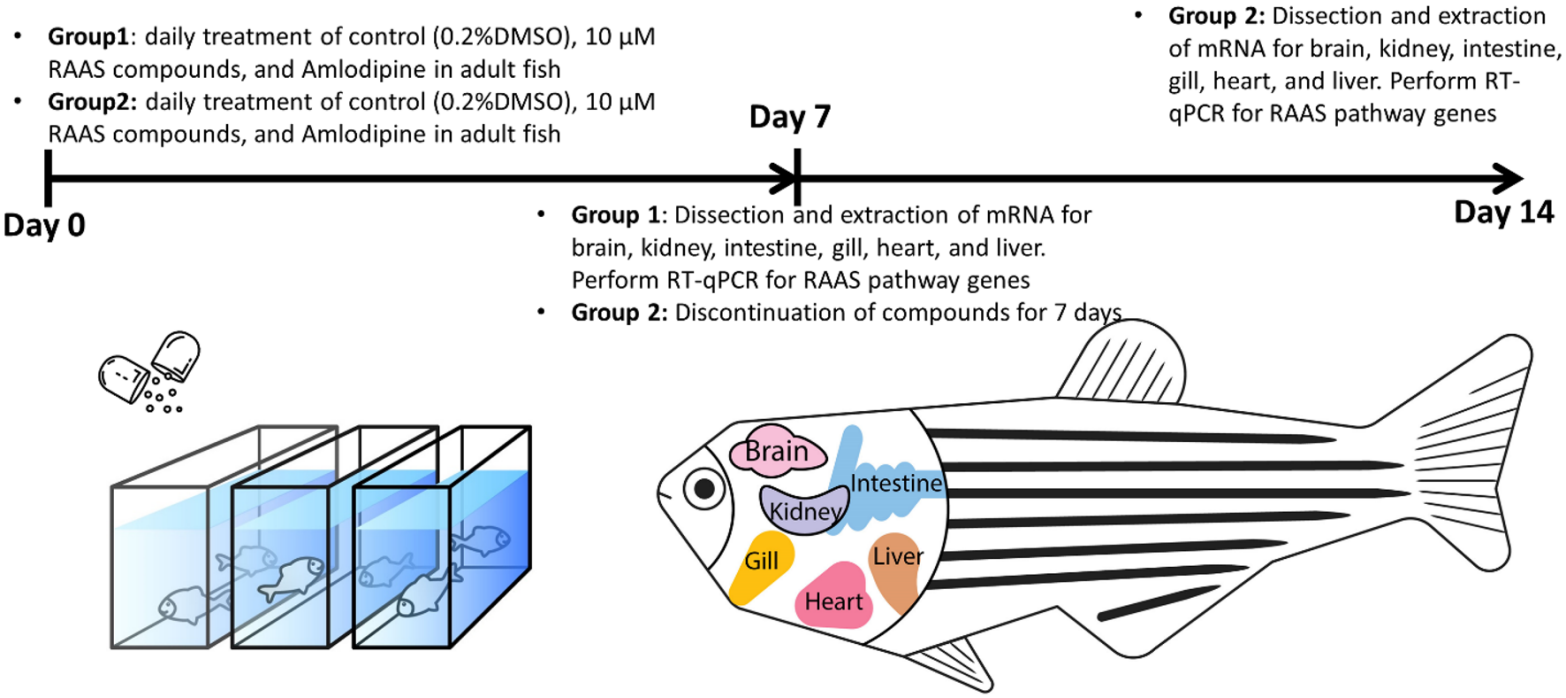
A schematic diagram for the experimental design for drug treatment and discontinuation. To determine mRNA expression of RAAS genes following one week of chronic antihypertensive drug administration, Group 1 consisted of zebrafish that were treated with 0.2% DMSO (control), aliskiren, olmesartan, captopril, and amlodipine daily for 7 days, then dissected for qRT-PCR analysis. Additionally, Group 2 consisted of zebrafish that were treated identically as Group 1 for 7 days, but then drug administration was discontinued for 7 more days before dissection and qRT-PCR analysis. The extracted organs include the brain, kidney, intestine, gill, liver, and heart. Six biological replicates (3 males and 3 females) were used for each compound (or vehicle) in each group.

**Figure 3:**
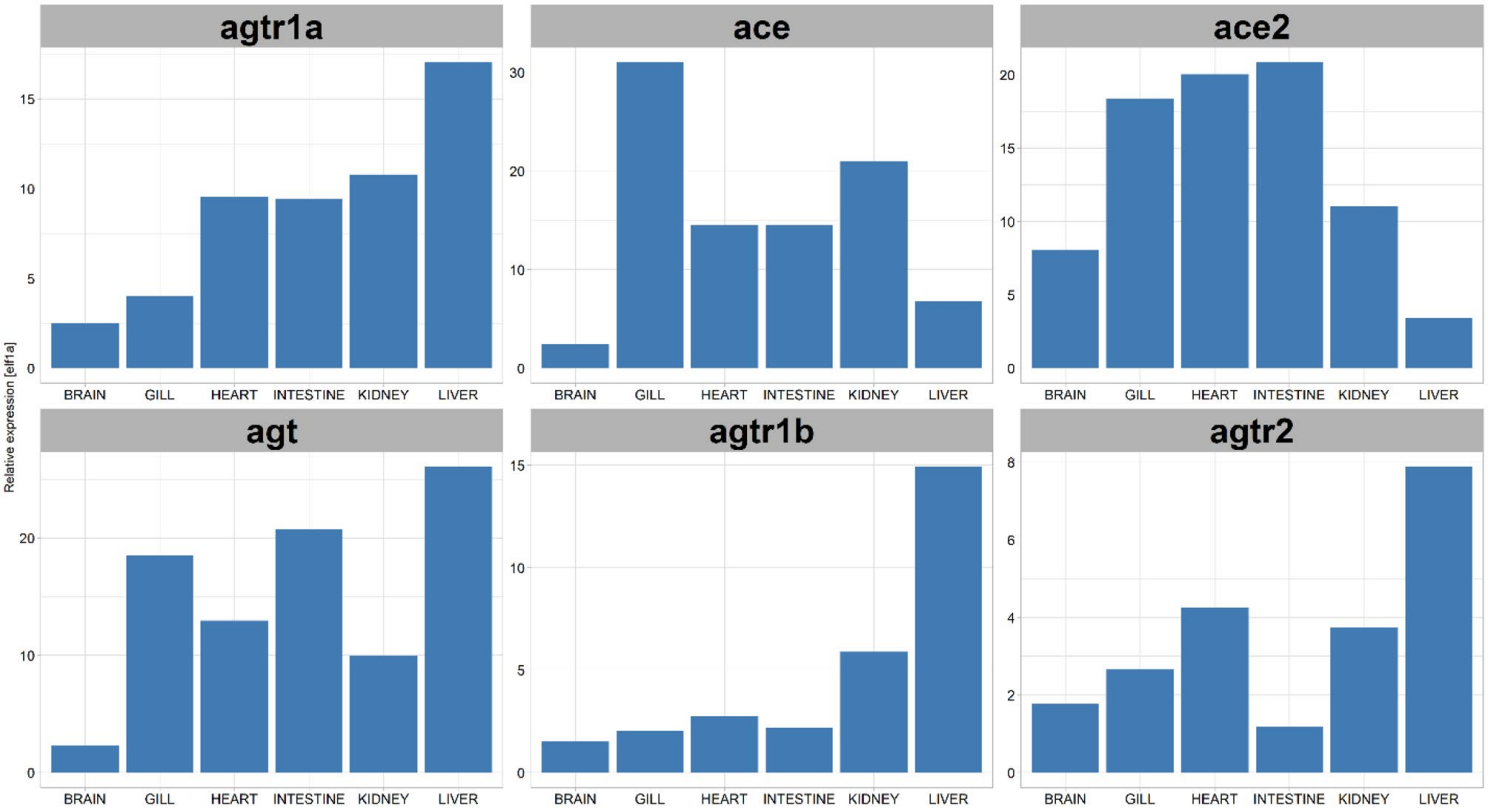
RAAS pathway gene expression displays tissue-specific enrichment. The relative expression levels of *agtr1a*, *ace*, *ace2*, *agt*, *agtr1b*, and *agtr2* to *elf1a1* in different organs of wild type adult zebrafish (n = 6). *agt*: Angiotensinogen, *agtr1b*: Type-1B angiotensin II receptor, *agtr2*: Type-2 Angiotensin II Receptor, *agtr1a*: Type-1A angiotensin II receptor, *ace*: Angiotensin-converting enzyme 1, *ace2*: Angiotensin-converting enzyme 2.

Major genes of the RAAS pathway and available small molecules that inhibit RAAS signaling are schematized (Fig. 1). To determine tissue-specific mRNA expression of RAAS genes in untreated conditions, adult zebrafish organs including the brain, gill (physiologically similar to the lung), heart, intestine, kidney, and liver were extracted from aged AB wildtype fish of 2–3 years old, to better mimic the life stage of COVID patients who might be taking these drugs (Fig. 2). After tissue homogenization and isolation of RNA, qRT-PCR was performed for angiotensinogen (*agt*), angiotensin II receptor type 1a (*agtr1a)*, angiotensin II receptor type 1b (*agtr1b)*, angiotensin II receptor type 2 (*agtr2)*, angiotensin converting enzyme (*ace)*, and Angiotensin converting enzyme 2 (*ace2)* (Fig. 3). The elongation factor 1 alpha (*elf1a1*) was used as the control housekeeping gene to quantify the relative expression of RAAS genes. There was no significant difference in RAAS gene expression levels between sexes for all the different organs (Fig. S1). For *ace2*, the gill, heart, and intestine showed higher expression levels of nearly 1.5-fold relative *to elf1a1*. The kidney, brain, and liver also showed *ace2* expression but at lower levels compared to *elf1a1*. The liver showed high expression of *agt* and the angiotensin receptors *agtr1a* and *agtr1b*. Trace amounts of *agtr2* expression was detected across all organs but showed lower levels relative to *elf1a1*. Together, while *ace2* expression is detected in all organs examined, it appears to be enriched in the gill, heart, and intestine.

### Treatment with RAAS inhibitors increases ace2 expression in a drug- and organ- specific manner

**Figure 4:**
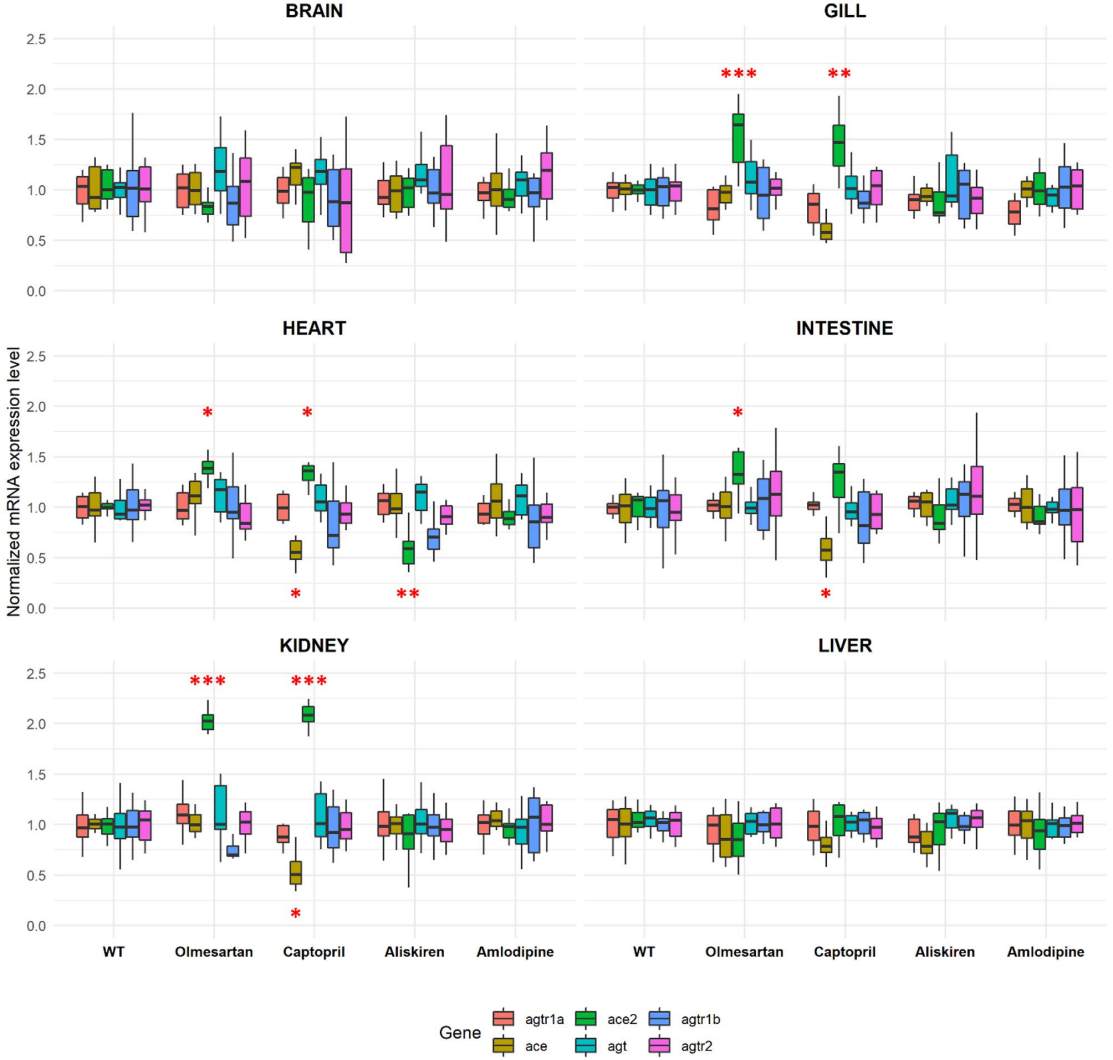
Tissue-specific up-regulation of *ace2* expression by RAAS inhibitors. The *ace2* expression was significantly increased in captopril and olmesartan treated groups compared to the DMSO control in the gill, heart, and kidney (n = 6 per gene-organ-drug combination, *p < 0.05, **p < 0.01, ***p < 0.001 unpaired t test). Olmesartan also increased *ace2* expression in the intestine (n = 6, *p* < 0.05, unpaired t test). Aliskiren treatment significantly decreased the *ace2* expression in the heart; captopril treatment decreased *ace* expression in the heart, intestine, and the kidney (n = 6, *p* < 0.05, unpaired t test). Olm: olmesartan, Cap: captopril, Ali: aliskiren, Amlo: amlodipine.

The transcript levels of the RAAS pathway genes were examined after 7 days of RAAS inhibitor or vehicle (0.2% DMSO) treatment (group 1) (Fig. 2) in aged AB wildtype zebrafish of 2–3 years old. Data were normalized to vehicle control for each gene-organ combination (Fig. 4). In addition to RAAS inhibitors, we also used amlodipine (a non-RAAS antihypertensive) as a comparison. Daily monitoring showed no physiological abnormalities, and all samples were included after the completion of 7-day treatment. Olmesartan treatment significantly increased *ace2* expression in the gill, heart, intestine, and kidney (*p* < 0.001 for gill, *p* = 0.012 for heart, *p* = 0.043 for intestine, *p* < 0.001 for kidney, unpaired t-test). Captopril increased *ace2* expression in the gill, heart, and kidney (*p* = 0.0087 for gill, *p* = 0.026 for heart, *p* < 0.001 for kidney, unpaired t test). Conversely, aliskiren treatment significantly decreased *ace2* expression in the heart (*p* = 0.021). In addition, captopril decreased *ace* expression in the heart, intestine, and kidney (*p* = 0.012 for heart, *p* = 0.037 for intestine, *p* = 0.026 for kidney, unpaired t test). The interaction coefficient between drug treatment, gene, and organ identified a total of 11 significant combinations at the 95% confidence interval (Supplemental Fig. 2). Thus, RAAS inhibitors increase *ace2* expression in a drug- and organ-specific manner: the ARB Olmesartan and ACE-I captopril commonly increase *ace2* expression in the gill, heart, and kidney. Olmesartan also increases *ace2* expression in the intestine. However, the renin inhibitor aliskiren decreases ace2 expression in the heart.

### The expression of ace2 is largely unchanged upon discontinuation of RAAS inhibitors for seven days

**Figure 5:**
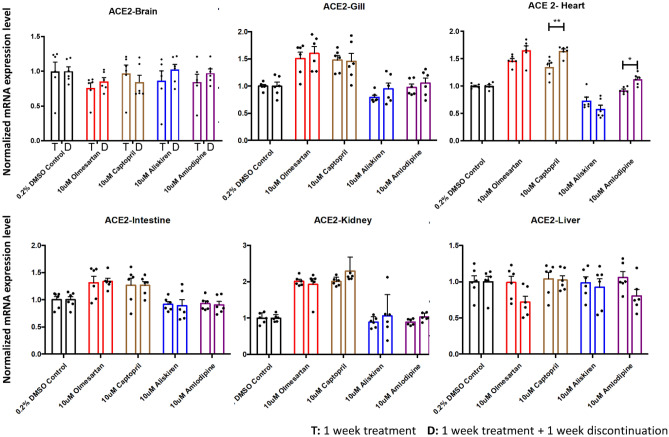
Comparison of *ace2* expression levels between treatment and discontinuation. The expression levels were compaired between the 1-week treatment group and 1-week treatment plus 1-week discontinuation group for different organs. The captopril and amlodipine treatment increased *ace2* mRNA expression in the heart 7 days after discontinuation compared to 7-day treatment (n = 6 per group; *p < 0.05, **p < 0.01, unpaired t test). Other organs showed no significant difference between treatment and discontinuation. T: 1 week treatment D: 1 week treatment + 1 week discontinuation.

For a second group of zebrafish (group 2), the fish were treated with 7 days of RAAS inhibitors, amlodipine, or vehicle control. The compounds were then discontinued for 7 days, followed by organ dissection and qRT-PCR analysis (Fig. 2) to determine whether discontinuation would have an effect on gene expression levels. When comparing the *ace2* expression between treatment (group 1) and treatment plus discontinuation (group 2), we found no significant *ace2* expression differences in the brain, gill, intestine, kidney, and liver. The elevated *ace2* expression in the gill, kidney, and the heart following 7-day olmesartan and captopril treatment was not significantly altered after 7-day discontinuation of these compounds. In contrast, discontinuing captopril and amlodipine treatment for 7 days significantly elevated *ace2* expression in the heart compared to 7-day treatment with these compounds (*p* = 0.0013 for captopril, *p* = 0.0419 for amlodipine, unpaired t test) (Fig. 5). The decrease in *ace2* expression with 7-day aliskiren treatment in the heart did not change following 7-day discontinuation. Together, 7-day discontinuation of RAAS inhibitors does not significantly alter *ace2* transcript levels.

## Discussion

In this study, we have determined that different RAAS inhibitors have varying effects on the *ace2* mRNA expression across different organs. We have also shown that adult zebrafish express all the major components of RAAS pathway genes that have been used in this study. Many of these gene expression profiles across zebrafish organs closely resembled that of the human organs. For instance, the angiotensin receptor type 1 (*AGTR1)* RNA in humans is predominantly expressed in the liver, kidney, adrenal glands, and adipose tissues^13^. For the zebrafish, the liver showed the highest expression of both *agtr1a* and *agtr1b* followed by kidney, heart, and intestine. The zebrafish kidney showed the highest *ace2* expression which is also the case of humans^13^. The RAAS gene expression in the brain showed minimal changes for both treatment and discontinuation. This could be because the antihypertensive drugs used in this study have very low bioavailability in the brain. While there are studies that show decreased ACE activity with oral captopril in the cerebral spinal fluid^14^, convincing evidence on whether these drugs cross the blood brain barrier remains unclear.

The gill, kidney, heart, and intestine showed significantly higher *ace2* expression when treated with an ACE-I and an ARB. It is known that ACE inhibitors such as captopril activate the ACE2/angiotensin-(1–7) /Mas receptor axis which could lead to the increase of *ace2* expression at the transcript level^15,16^. In a study conducted on Lewis rats it was shown that lisinopril, another commonly used ACE inhibitor, caused an increase in plasma Ang-(1–7), and increased cardiac *ace2* mRNA but did not affect the ACE2 protein expression^17^. Interestingly, *ace2* expression was decreased in the heart with 7-day aliskiren treatment. As a direct renin inhibitor, the pharmacological action of aliskiren is known to affect the AngII/ Ang1-7 signal axis^18^. In a diabetic neuropathy model of Sprague Dawley rats, it was shown that chronic administration of aliskiren decreased ACE2 expression in the kidneys and this decreased expression was also observed in another hypertension rat model^18,19^. As many patients who experience COVID symptoms have pre-existing cardiovascular comorbidities, it is common that these patients use RAAS inhibitors chronically. In our zebrafish model we have observed that the 7-day discontinuation did not alter the *ace2* expression in most organs examined compared to the 7-day treatment groups. In this case, an abrupt discontinuation of RAAS inhibitors, particularly if already being used for other indications, would not be beneficial in managing COVID symptoms.

One side of the ongoing debate on use of RAAS inhibitors in the COVID-19 setting is toward continuing the medication based on the large cohort studies that find no association between the use of RAAS inhibitors and susceptibility to SARS-CoV-2 infection^20–22^. Studies are also trying to understand whether the increase in *ace2* can potentially be linked to protective benefits. Clinical trials have been conducted on initiating losartan, another commonly used ARB, for COVID-19 hospitalized patients (NCT04312009). The downregulation of ACE2 with COVID-19 could lead to an increase in ACE activity resulting in damage to the alveolus and lead to acute respiratory failure which could warrant the use of RAAS inhibitors^23^. A meta-analysis of evaluating patients with COVID-19 showed a significantly lower risk of severe adverse events among patients who received ACE-Is or ARBs with implications on the protective benefits^21^. In another study it has been shown that the RAAS imbalance through angiotensin II or ACE2 blockage shows clinical manifestations closely resembling COVID-19^24^.

Although we have chosen three compounds with mechanism of actions that target different parts of the RAAS pathway, the *ace2* expression could also be different based on the chemical structure, pharmacokinetics, and receptor affinities. Olmesartan medoxomil is a prodrug that requires hydrolysis to the active form. Olmesartan medoxomil undergoes hydrolysis to its active form by esterases during absorption in the gastrointestinal tract^25^. Valsartan and lisnopril, other commonly used RAAS inhibitors, are active drugs that do not undergo extensive metabolism and are excreted unchanged in the urine. Losartan, another ARB, has nearly tenfold greater selectivity compared to olmesartan^26^. Whether these differences in molecular effects have implications in the clinical setting is not clear. As we have tried to mimic the study to be a chronic treatment model, the treatment and discontinuation duration was chosen as 7 days based on other studies that involved continued drug treatment for pharmacological studies in zebrafish^27,28^. While this is certainly not a direct comparison to the long duration that many hypertension patients have been taking chronically for years, based on the pharmacokinetic properties of the RAAS inhibitors used in the study, the direct uptake of the drugs in the water bath should reach steady state at the target tissues during the treatment duration.

In conclusion, our study uncovers that RAAS inhibitors can influence the RAAS pathway gene expression in an organ-specific manner in zebrafish. The organs that were most sensitive to changes in *ace2* expression include the gill (physiologically similar to the lung), heart, intestine, and kidney, which are all known target sites of COVID-19 leading to clinical manifestations. Furthermore, the expression levels after 7 days of discontinuation did not show remarkable changes in gene expression. Although more studies need to be done to understand how these gene expression profiles translate at the protein level, our study provides new insights into the transcript level modulation of RAAS pathway genes with RAAS inhibitor treatment. This basic knowledge lays foundation for deciding the use of RAAS inhibitors in the context of COVID-19.

## Methods

### Zebrafish husbandry and ethics statement

For all experiments, the wild type of the AB strain adult zebrafish was used in this study. All experimental protocols and procedures were approved by the University of California San Francisco Laboratory Animal Resource Center and Institutional Animal Care and Use Program. All study procedures were performed in compliance with the ARRIVE guidelines. The zebrafish were raised on a 14:10 h light/dark cycle and maintained in the zebrafish facility in accordance with the University of California San Francisco Institutional Animal Care and Use Committee standards.

### Sample setup, drug treatment, and discontinuation

The adult zebrafish were housed in 1-L tanks separated based on what compound they receive and the treatment group (group 1) and treatment plus discontinuation group (group 2). For each group, three aged males and females between 2–3 years old were selected to control for sex and age distribution per compound (or vehicle control). Each tank was filled with 500 mL of system water along with the dissolved compounds. All samples were treated with a concentration of 10 μM olmesartan medoxomil, captopril, aliskiren hemifumarate, or amlodipine besylate. The vehicle control was treated with 0.2% DMSO. The tanks were changed to fresh system water and compounds daily. The compounds olmesartan medoxomil, captopril, aliskiren hemifumarate, amlodipine besylate were obtained from Sigma-Aldrich (cat #144,689–63-4, 62,571–86-2, 173,334–58-2, A5605).

### Extraction of total RNA and cDNA synthesis from adult zebrafish organs

The adult zebrafish were treated with 2 µg/mL of tricaine for sedation and the brain, kidney, heart, intestine, liver, and gill were dissected. Total RNAs were prepared from isolated adult tissues of zebrafish using Trizol reagent (Invitrogen cat no 15596026) by homogenization and purified using RNeasy Mini Kit (Qiagen cat no 74104). cDNAs were synthesized from 1 µg of purified RNA using SuperScript® IV First-Strand Synthesis System for RT-PCR (Invitrogen cat no 18091050) and used as templates.

### Quantitative polymerase chain reaction (qPCR) analysis

The primers for the qPCR were designed with the NCBI primer blast. The primer sequences and the ensemble ID for each gene were listed in Table 2. The PCR product size was designed to span 120 to 200 bp with low self 3′-complementarity score. Different primer designs were initially validated with gel electrophoresis to determine the optimal forward and reverse pair with specific amplification of the desired sized products. qPCR was performed using Applied Biosystems SYBR Green PCR Master Mix (Thermofisher cat no 4367659) and the ABI7900HT (Applied Biosystems machine cat no 4329001). Cycling conditions were 95 °C for 10 min, [95 °C for 15 s, 60 °C for 1 min 40 cycles], 20 °C for 2 min. Each sample was run with triplicates along with the positive control (elf1a1) in the top row of the MicroAmp Optical 384-Well Reaction Plate (Applied Biosystems cat no 4309849). The Ct values were exported and ΔCt values were calculated to compare relative expression of the genes of interest to the *elf1a1* control.

**Table 2:** Summary of RAAS pathway genes, RT-qPCR primer sequence, and Ensembl ID used for the RT-qPCR analysis.

Gene Name	Gene symbol	Function	Primer Sequences (5′- > 3′)	Transcript ID	Product length
Angiotensin II receptor, type 1a	*agtr1a*	G protein-coupled receptor for angiotensin II	F: CATCCGTGGGACCCATTTCA	ENSDART00000021528.7	154
			R: GCAGTAGCACGTGAGGATGA		
Angiotensin II receptor, type 1b	*agtr1b*	G protein-coupled receptor for angiotensin II	F: TTCATGCCGTTTGGCTCAGA	ENSDART00000066834.5	200
			R: GGTCCTCGCTCATTGCTGAT		
Angiotensin converting enzyme	*ace*	Metallopeptidase involved in the conversion of angiotensin I to angiotensin II	F: GAGCCAATCCTGGCTTCCAT	ENSDART00000114637.4	133
			R: CCGATGACGCTGAGAGTGAC		
Angiotensin converting enzyme 2	*ace2*	Transmembrane protein catalyzing angiotensin II hydrolysis. Main entry point for coronavirus	F: CTGATGCCTGTCTTCCAGCA	ENSDART00000003712.8	141
			R: TTTCATCCCAACCCTGCTCC		
Angiotensinogen	*agt*	Precursor molecule for angiotensin I and the substrate for renin produced in the liver	F: GGCTTCGACACCTCAAGGAA	ENSDART00000010918.5	192
			R:ACACCACCTTGTTGAGTACCTTA		
Angiotensin II receptor, type 2	*at2*	G protein-coupled receptor. Regulation of aldosterone secretion	F: GTTCACGAACATCAGAACTCCC	ENSDART00000051532.5	188
			R: TGAGGCTGTAAAAGGCAGGG		
Elongation factor 1 alpha 1	*elf1a1*	Housekeeping gene for RT-qCPR	F: CTTCTCAGGCTGACTGTGC	Adopted from McCurley et al^29^	–
			R: CCGCTAGCATTACCCTCC		

### Statistical analysis

The mRNA expression from the qRT-PCR was processed by the 2^−ΔΔct^ method in comparison to the *elf1a1* housekeeping gene. The histograms in the study are represented as means ± standard error of the mean (SEM). The box and whisker plot are represented as medians with first and third quartile ranges. The gene-organ-drug interaction was examined with the R ‘interactions’ package and multiple regression model. The comparison between drug treatment and discontinuation was compared with an unpaired t-test. The 95% confidence interval of the gene-drug-organ interaction was analyzed with the R program.

## Supplementary Information

Supplementary Information.
