# Supplementary material for: The effect of renin–angiotensin–aldosterone system inhibitors on organ-specific ace2 expression in zebrafish and its implications for COVID-19
Source: Sci Rep. 2021 Dec 8;11:23670. doi: 10.1038/s41598-021-03244-5 (PMC8655050; doi:10.1038/s41598-021-03244-5)
Supplement: Supplementary file 1 — Supplementary Information. [file 41598_2021_3244_MOESM1_ESM.docx]

**
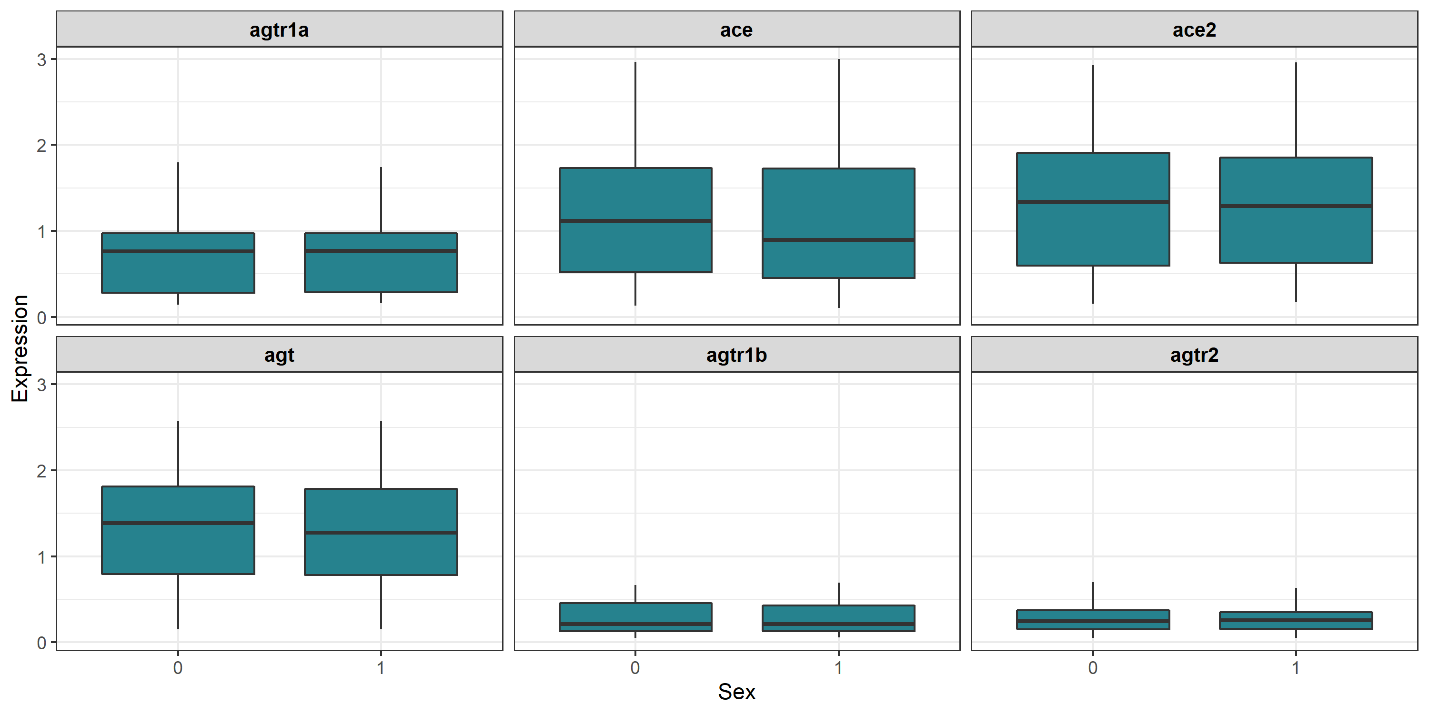
**

**Supplemental Figure 1. Wildtype zebrafish shows no difference in sexes for mRNA expression.** The expression levels of *agtr1a*, *ace*, *ace2*, *agt*, *agtr1b*, *agtr2* for the wildtype showed no significant difference between sexes. 0: male 1: female (n=6 per gene-sex combination; unpaired t test)


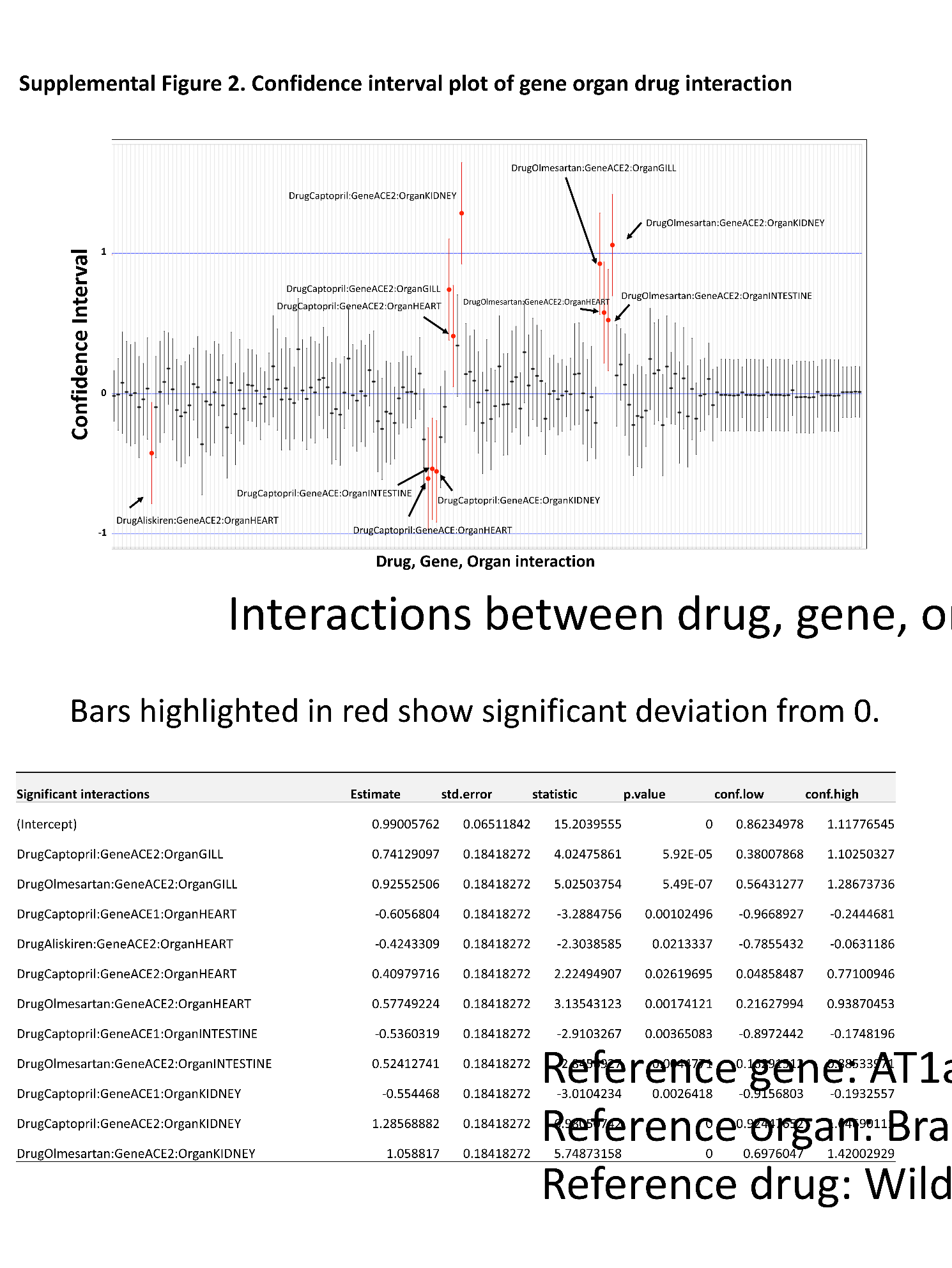


**Supplemental Figure 2.** **Confidence interval plot of gene organ drug interaction.** A total of 11 drug-gene-organ combinations showed significant difference at the 95% CI.
